# Use of Ultra Wide Band Real-Time Location System on Construction Jobsites: Feasibility Study and Deployment Alternatives

**DOI:** 10.3390/ijerph17072219

**Published:** 2020-03-26

**Authors:** Waleed Umer, Mohsin K. Siddiqui

**Affiliations:** 1 Department of Construction Engineering & Management, King Fahd University of Petroleum & Minerals, Dhahran 31261, Saudi Arabia; 2 Department of Civil and Environmental Engineering, University of Delaware, Newark, DE 19716, USA; mohsin@udel.edu

**Keywords:** ultra wide band (UWB), real-time location systems (RTLSs), construction sites, sensors, location positioning

## Abstract

Ultra wide band (UWB)-based real-time location systems (RTLSs) have been widely adopted in the manufacturing industry for tracking tools, materials, and ensuring safety. Researchers in the construction domain have investigated similar uses for UWB-based RTLSs on construction jobsites. However, most of these investigations comprised small-scale experiments using average accuracy only to demonstrate use cases for the technology. Furthermore, they did not consider alternative deployment scenarios for practically feasible deployment of the technology. To overcome these limitations, a series of experiments were performed to study the feasibility of a commercially available RTLS on the construction jobsites. The focus of the work was on feasibility in terms of accuracy analysis of the system for a large experimental site, the level of effort requirements for deployment, and the impact of deployment alternatives on the accuracy of the system. The results found that average accuracy was found to be a misleading indicator of the perceived system performance (i.e., 95th percentile values were considerably higher than average values). Moreover, accuracy is significantly affected by the deployment alternatives. Collectively, the results arising from the study could help construction/safety managers in decision making related to the deployment of UWB-based RTLSs for their construction sites.

## 1. Introduction

Technology-enabled jobsites have the potential to fundamentally alter the business processes on construction jobsites. Data sensing and analysis techniques can enable decision makers to make informed decisions in support of the management of day-to-day construction operations. Recent research works have identified construction safety and productivity as two main knowledge areas where data sensing and analysis can have a large impact [1]. Information and data requirements in support of these knowledge areas mainly consist of locations of different assets and materials. Various technologies have been reported in the literature to ascertain these locations with different levels of accuracy and timing [2].

Manual onsite data collection is time consuming, prone to errors, and a tiresome activity. Real-time location systems (RTLSs) have been used to collect accurate location data in real-time [3]. Real-time location data can help in a number of ways, for example, (1) preventing accidents on a construction site [4], (2) preventing potential collisions between equipment and manpower, and (3) fall from height accidents [5]. Specifically for preventing accidents on construction sites, RTLS can be used to closely monitor the location and movements of workers and machinery on construction sites, which could be used to alert workers when they are in the near vicinity of a hazardous area or when moving machinery is too close to workers [6,7]. Similarly, RTLS could also help where contact collision between a worker and material poses a serious risk [8]. Likewise, collisions among construction equipment mostly due to unawareness of precise location are also common, which could effectively be reduced by using RTLS-based alerting systems [9]. Besides, material and equipment tracking is also of vital importance. The losses due to theft of equipment and tools were estimated to be $1 billion in the United States for the year 2001 [10].

Diverse location sensing technologies have been reported in the literature and their limitations have been discussed a number of reviews [11]. The technologies studied include radio frequency identification (RFID), embedded sensors, global positioning system (GPS), Flash LADAR, LASER scanner, high-resolution video camera, digital photogrammetry, and wireless communication. Vision-based sensors require line of sight for accurate performance and are limited in their working range [12]. Communication-based technologies provide longer working ranges, but are prone to signal interference from other sources and environmental factors prevailing on jobsites. Ultra wide band-based RTLSs have been shown to be more accurate as compared with traditional RFID- and GPS-based sensing for determining locations on jobsites [13].

A more recent review by Zhang et al. [14] examined the use of sensor-based technologies to improve construction safety. Research on the potential use of UWB remains an active area, where researchers have reported proof of concept studies in different construction-related settings. Researchers have explored the accuracy with which UWB can operate on construction jobsites in different settings [3,15,16,17,18,19,20,21]. Knowledge and operation domains studied for UWB include safety and productivity of construction resources [22,23,24,25,26,27]; tracking and visualization of assets including workforce, equipment, and materials [28,29,30,31,32,33]; safety of critical equipment such as cranes [34,35]; and educational purposes [36].

### 1.1. Literature Review

The use of ultra wide band (UWB)-based real time location system (RTLS) has been frequently reported in the manufacturing domain. Large manufacturing and operating concerns have used these systems for diverse purposes including automatic tool adjustment on assembly lines, theft prevention systems, emergency evacuation planning and management, warehouse management, and management of buses in real time in a yard [37,38,39]. RTLS has also been used to track humans to improve patient care management systems [40]. The static nature of the operational areas is a common factor among all these deployments. Although some calibration free systems are available for indoor positioning, most UWB-based RTLSs require infrastructure support [41]. A typical system deployment approach includes a site survey to identify optimal sensor (signal receiving device) locations, deployment of sensors and cabling, and calibration leading to the beneficial use of the system [42]. Performance remains consistent during the deployed life of the system and accuracy depends on both the operating environment and the diligence with which the specific deployment actions were performed. Degradation of operational performance may occur over time, but it is usually overcome by recalibration of the system.

Although some construction operations (such as prefabrication) utilize similar manufacturing-oriented processes, in general, the construction industry environment is quite different from the static industrial settings. Construction sites present additional challenges owing to the dynamic environment that requires a constant response to changes. With the ongoing construction progress, the beneficial use of RTLS in these settings would require frequent alterations and re-calibrations that can potentially have an impact on the performance and accuracy of the system. Prior to identifying the related knowledge gap in Section 1.2, the following sections review the relevant literature in the construction industry, which summarize studies in chronological order of publication.

#### 1.1.1. Performance of UWB-Based RTLS

The RTLS uses time difference of arrival (TDOA) and angle of arrival (AOA) techniques to localize a target. Although the system can localize the target using AOA data only, TDOA enhances the accuracy and credibility of the location data collected by the system. Sometimes TDOA is not utilized because it requires additional wiring to transmit the data, which is undesirable on complex and dynamic locations such as construction sites. However, most research work mentioned in this section relied on using both TDOA and AOA information to maximize the accuracy of the system.

Mok et al. [21] reports on a comparison of the performance of different communication-based systems for locating assets. Small-scale experiments were conducted in a lab setting where it was determined that, among different systems, UWB-based RTLS was the most accurate system. Outdoor experiments were performed in a tunnel, resulting in an average accuracy of up to 1 m. The researchers concluded that cable-based systems may not be suitable for complex settings like construction sites.

In a separate study, untethered UWB-based RTLS was tested in which wireless connection for time difference of arrival (TDOA) data transmission was established in place of wired connections [18]. The experimentation results showed a slight decrease in the accuracy of the location data for the untethered network as compared with the cabled network. Both static and dynamic tests were performed. The researchers reported that the human body adversely affects the accuracy of UWB-based RTLS. Furthermore, it was found that an increase in the height of the tag (signal emitting device) from ground level to around 1 m showed an improvement in the accuracy, as it improved the line of sight between the sensors and the tags. Finally, this study concluded that, with the untethered networking of UWB-based RTLS, accuracy of up to 50 cm in static conditions and 65 cm in dynamic conditions in a dense construction site can be achieved.

In another study, UWB-based RTLS was tested in an outdoor construction environment, where different update rates were used for mobile and static asset tracking [17]. The researchers reported an average accuracy of 2 m at a distance of 270 m from the sensors. It was asserted that such an accuracy would be sufficient for material tracking in a storage yard.

Similarly, a study conducted dynamic and static experiments to examine the performance of a UWB-based RTLS in an experimental setup [16]. A robotic total station (RTS) was used to establish the ground truth. The static experiments were performed in an outdoor area of 20 m by 10 m, where the researchers studied the effect of changing the transmitters’ height and calibration precision on the accuracy of the RTLS. An increase in the transmitters’ height yielded better results for accuracy. For calibration precision, better calibration (robotic total station as compared with GPS with an accuracy of 20 to 30 cm) also led to better accuracy. For dynamic experiments, which were carried out in a large construction laydown yard, the researchers concluded that the achieved average accuracy was sufficient for tracking the machinery and material locations.

Maalek and Sadeghpour [5] performed a number of experiments that were designed to test the accuracy of a commercial off-the-shelf UWB-based RTLS. The study explored the effect of different factors on the accuracy including the multipath effect, the presence of metal surfaces, the effect of movement, number of simultaneous tags in operation, and using angle of arrival (AOA) information only. Distance root mean square (DRMS) was used to assess the 2D accuracy of the system, while mean radial spherical error (MRSE) was used to investigate the 3D accuracy of the RTLS. An average accuracy of 0.30 m to 0.6 m was reported in different settings, and it was concluded that the system could provide sufficient accuracy in a multipath environment with the presence of metals for asset tracking purposes on construction sites. Besides, the system was capable of tracking the resources even when only the angle of arrival method was used. As mentioned earlier, the key detriment of using the TDOA comes from the wiring required, which can be a challenge in dynamic environments [21]. Overall, the studies reported in this section relied on average accuracy as a measure of performance and recommend the use of the RTLS for tasks such as material tracking and site planning.

#### 1.1.2. Domain Applications in Construction

Studies from the construction domain show that the use of RTLS may be extended to other domain functions such as safety, material tracking, and productivity analysis. The UWB-based RTLS may be used in conjunction with other sensing techniques to improve safety [24]. Recent research has highlighted that blended technologies are helpful in managing the work site more effectively [29].

In a study, using UWB-based RTLS, the movement of workers was observed to avoid obstacles and to generate a safe path on a construction site [25]. Convex hull algorithm was used to identify the obstacle contours and to encompass the obstacle with a convex polygon and, consequently, the shortest and safest paths were identified.

In another study, a safety system was developed to ensure real-time collision free paths for crane movements using UWB-based RTLS [4]. Rapidly-exploring random trees (RRT) and dynamic rapidly-exploring random trees (DRRT) algorithms were used for path planning and re-planning, respectively, in real time. By combining visualization and decision making modules with the real-time location data, a safety system was presented that was tested on laboratory prototypes [34].

RTLS has also been used for tracking worker activities on tasks such as painting and welding that cannot be tracked easily using existing progress tracking systems [30]. The data from RTLS were integrated with CAD information to generate as-built drawings. A fusion of emerging visualization technologies such as virtual reality (VR) and RTLS has also been explored in the literature [26]. The information gathered was used to enhance the situational awareness and highlight spatial conflicts on the jobsite.

In an interesting study, RTLS information was combined with a commercially available physiological status monitoring (PSM) system to integrate the physiological status of a construction worker and location data on a job site [27]. This information was used to monitor the physiological health of a worker as he performed his job task, which included lifting the material, bending, and squatting.

Other innovative approaches on data fusion have also been reported in the literature, including an intelligent concrete consolidation tracking system for vibration tool, which could visualize the location, time, and depth of the vibration done [32]. This helped in the identification of the spots that need mitigation actions for freshly poured concrete.

### 1.2. Knowledge Gap

Existing research sparingly discusses deployment protocols or practical accuracy requirements for location systems for adequate support of knowledge domain functions. Significant improvements can be achieved for material tracking even with lower levels of accuracy; however, feasible deployment of safety alert systems requires a higher level of accuracy of the underlying location system. Researchers in the construction domain have used average accuracy during small-scale experiments as an indicator of the performance of commercially available ultra wide band (UWB) systems [14]. Average accuracy may be suitable for productivity related operations, however, the authors believe that the average measures are deficient for construction safety purposes because “average” implies that, generally, 50% of the values will be greater than the average value, which may consequently exaggerate the accuracy performance. Hence, more stringent metrics are required to ensure safety on a construction site. Additionally, most deployments reported in the literature and practice are case studies with coverage extending to only a portion of the jobsite or test area with sensors placed in a recommended geometry. Hence, there is a need to explore the deployment on larger sites with alternatives geometry of the sensors too, in order to better comprehend the performance and benefits of UWB-based RTLS.

To fulfill this knowledge gap, this study used a higher threshold of accuracy performance (95th precentile) in a large experimental setting (approximately 2000 m^2^) to demonstrate the feasibility of the use of a UWB-based RTLS on construction jobsites. Unlike manufacturing domains, where a system is deployed once, calibrated, and then used for extended periods, the authors envision that practical field deployments of UWB systems on actual construction jobsites would entail frequent repetitive deployment of sensing systems a number of times. A series of experiments were carried out on an open jobsite for multiple scenarios to replicate the conditions on the jobsites. The scenarios were designed considering the shortcomings mentioned in the literature. These scenarios included investigating accuracy for a single tag versus multiple tags, different possibilities of location placements of the sensors, and static and dynamic experiments. Accuracy (2D and 3D) parameters were compared with a baseline deployment, which was done as per manufacturer guidelines. The average accuracy and 95th percentile were chosen as accuracy parameters. Finally, the results were analyzed by merging accuracy heat maps and field of view (FOV) images, leading to the conclusions of the research work. Accordingly, this paper proceeds as follows. Section 2 delineates the material and methods employed for this research, which is followed by the results in Section 3. Discussions are made in Section 4 and, finally, conclusions are drawn in Section 5.

## 2. Materials and Methods 

**Figure 1:**
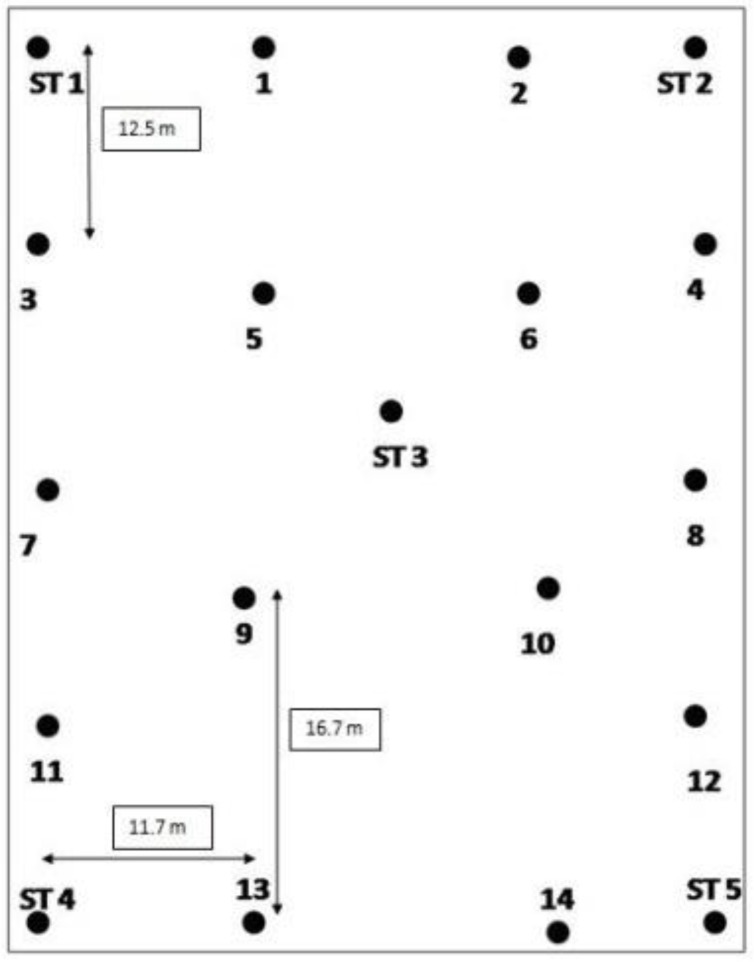
Layout of the experimental site. Note: ST 1 to 5 refer to the established benchmark stations. Deployment points 1 to 14 were fixed for tag deployment.

To explore the problems identified in the knowledge gap, a series of experiments were designed to test the RTLS under different sensors’ configuration setups on an open site. The dimensions of the site were around 40 m x 55 m. Figure 1 shows the layout of the area where the experiments were performed. Nineteen station points were established with the help of a laser scanner. Bronze benchmarks were installed in the ground at the site to repeat the experiments with or without altering the experimental conditions. These benchmarks are referred to as stations (ST 1 to 5) in Figure 1. Using this setup, the authors were able to achieve resection with a maximum error of 4 mm. The figure also depicts the spread of the station points on the experimental site. Sensors employed were from Ubisense (7000 series) with dimensions of 20 cm x 14 cm x 9.5 cm, whereas tags were 38 mm x 39 mm x 16.5 mm in dimensions.

### 2.1. Deployment Protocols

Following are the main steps involved in the deployment of a typical UWB-based real-time location system (RTLS) on construction jobsites. For each site, some minor steps may also be included, which will differ as per deployment of the system under different conditions.

#### 2.1.1. Layout and Cabling

This step involves deployment of the system’s hardware on the construction site. Firstly, the location of sensors has to be decided based on the coverage required for the site, access/availability of the locations on the site, site’s work environment, and other factors (such as sources of signal interference). The existing structures and infrastructure on the site will dictate whether the sensors can be deployed on fixed surfaces or whether temporary deployment apparatus (such as tripods) are to be used. Fixed surfaces such as walls are preferred over tripod stands because walls are firm and stable and are less prone to vibrations and winds on the construction sites. Furthermore, walls are usually safer than tripod stands as cranes and other mobile equipment may hit the tripod stands because their location is transient and may not be known to them. Tripod stands may fall, causing damage to the costly sensors. For the actual physical installation of the sensors, wall mountings are to be installed on fixed surfaces or the sensors can be fixed to the stem of the tripod using “U” shaped hooks.

Wired connections are necessary between the different sensors for utilization of the RTLS using the TDOA method. Wires are needed to be run among the sensors and from a master sensor to the computer running the software platform. A master sensor is that sensor that has been designated as a reference for time difference of arrival (TDOA) measurements. Power can either be provided to the sensors through DC supply or by power over ethernet (POE). POE was used in this study. Time and cost requirements will vary from site to site depending upon the area to be covered and distinctive site characteristics for each construction site (such as congestion, obstacles in sight of view, and the presence of nearby electronic equipment).

#### 2.1.2. Orientation and Survey

The accuracy of the system depends on the accurate survey of the sensor positions and reference points after the cabling and layout have been done. The coverage of the UWB-sensor used in our setup had a field of view of 120° in the horizontal plane and 100° in the vertical plane. The effective sensing range of the sensor also varies considerably based on the site characteristics. After the orientation adjustment, the survey needs to be done in order to know the location of the sensors. This can be done using various available surveying equipment like a total station or laser scanner. The man-minutes effort required depends on the number of sensors, site characteristics, and the efficiency of the surveying equipment. Additional man-minutes may be consumed if a higher level of accuracy is desired in this process.

#### 2.1.3. Calibration

There are various methods of calibration that can be adopted as recommended by the RTLS manufacturer. The calibration technique used in our setup involved placing a tag in a fixed central known position. Individual sensors are then calibrated with the designated master sensor to determine the pitch, yaw, roll, and cable offset for all the sensors. The accuracy provided by the system is heavily dependent on this calibration process. For construction sites, it is advisable to calibrate the system using multiple test points. Consistent resultant outputs of these calibrations indicate a good system setup.

### 2.2. Experimental Protocols

**Figure 2:**
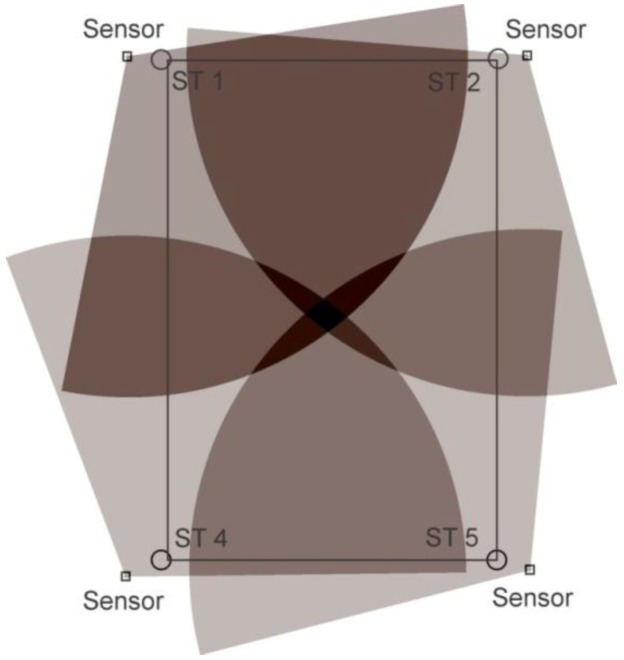
Full site configuration.

**Figure 3:**
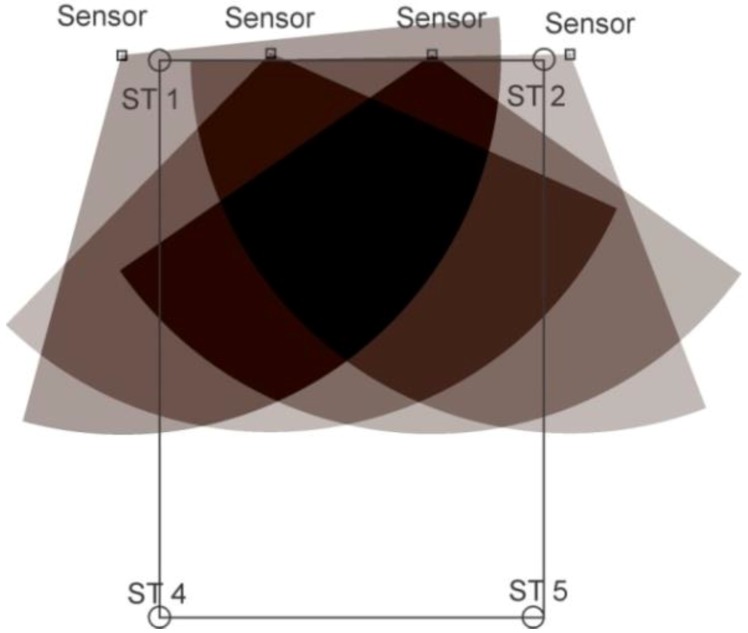
Offsite setup configuration.

**Figure 4:**
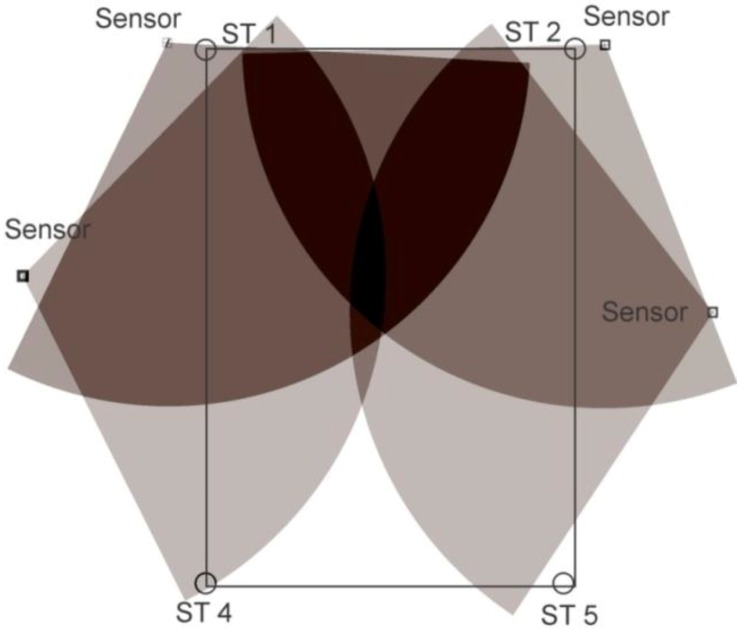
Partial site access configuration.

For static tests, tags were deployed at the station points for one minute; location data readings were noted and, finally, they were removed. For some of the experiments, a single tag was deployed one by one at all station points and, for the others, all of the station points were occupied by the tags simultaneously. Three setups were arranged for the knowledge gaps discussed earlier using various configurations, namely, full site access, offsite setup, and partial site access. These three setups are elaborated in the sections below. Figure 2, Figure 3 and Figure 4 shows the actual RTLS configuration for these setups. These figures show the actual positioning of the sensors relative to the station points, orientation, and field of view (FOV) of the sensors. To plot the FOV images for different scenarios, the roll, yaw, and pitch of all of the sensors were noted down from the experimental scenarios performed. Furthermore, the exact locations of the sensors with reference to the test field were obtained. Finally, this information was plotted using a graphics software while considering the FOV to be 110 degrees in the horizontal plane (as per the specifications of the RTLS).

In addition to the things discussed above, the filled arcs initiating from the sensors show the coverage provided by each of the sensors. For ease, the range of each of the sensors was considered to be 35 m, which may differ from the actual range of the sensors. It is important to note that better coverage is available where a place is covered by multiple sensors. The dark color in the center as compared with the sides of the site illustrates the same concept (Figure 2, Figure 3 and Figure 4).

### 2.3. Full Site Access 

The first scenario was termed as full site access, in which the system was installed as per the manufacturer’s specification. This setup was considered as a baseline for comparison between other setups. This cell configuration is considered for site conditions where full access to the site is available. Four sensors were spread in the corners. This fulfills the requirements for optimum accuracy at the construction site. This configuration and FOV are shown in Figure 2. 

### 2.4. Offsite Setup

This cell configuration is considered for the cases where the access to the construction site is either not feasible or not granted. The sensors were placed outside or at the one boundary of the construction site. This led to the scenario where real-time location data were collected offsite. The setup is shown in Figure 3. The advantage of such a setup is that, physically, no RTLS infrastructure has to be deployed on the construction site. This means there will be no cabling, no interruptions, and no safety hazards for the deployment of the RTLS on the work site. Other setups may cause accidents, causing damage to the construction assets or the RTLS.

### 2.5. Partial Site Access

This cell configuration is considered for the cases where the full access for the site is not available. The sensors were deployed on the partial site. The setup is shown in Figure 4.

Distance root mean square (DRMS) was used to assess the accuracy for 2D experiments, whereas mean radial spherical error (MRSE) was used to assess accuracy for 3D experiments. Both metrics, average accuracy and 95th percentile, were computed. MRSE and DRMS allow to combine precision and offset in one single value to represent accuracy [43].

Mathematically,

(1) $$DRMS=\sqrt{\frac{\sum_{i=1}^{n}(x_{i}-x_{actual)}{}^{2}}{n}+\frac{\sum_{i=1}^{n}(y_{i}-y_{actual)}{}^{2}}{n}}$$

$$MRSE=\sqrt{\frac{\sum_{i=1}^{n}(x_{i}-x_{actual)}{}^{2}}{n}+\frac{\sum_{i=1}^{n}(y_{i}-y_{actual)}{}^{2}}{n}+\frac{\sum_{i=1}^{n}(z_{i}-z_{actual)}{}^{2}}{n}}$$

where n is the total number of readings taken at a particular station point. x_i_, y_i_, and z_i_ are location coordinates for the i^th^ reading provided by the sensors. x_actual_, y_actual_, and z_actual_ are actual coordinates of that particular point already available using the laser scanner survey of the site.

Two variations for static experiments were performed:

1. A single tag deployed at different station points versus all tags deployed at once, as the number of tags being tracked has an impact on the accuracy;
2. All tags deployed at once with AOA only versus with both AOA and TDOA, that is, wireless setup versus wired setup, respectively.

**Figure 5:**
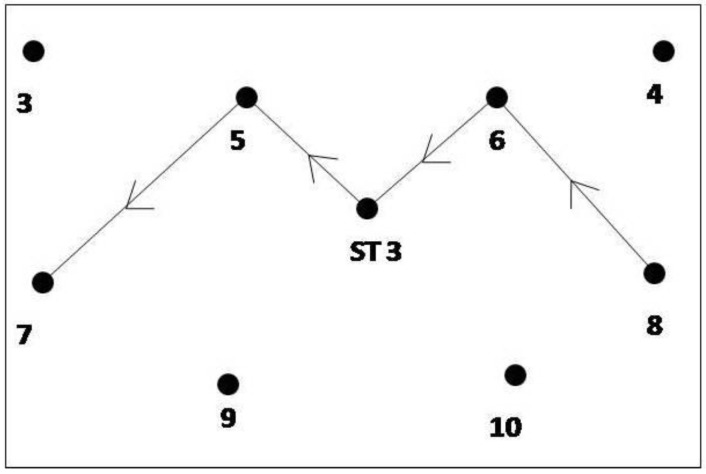
Layout for dynamic experiments.

For dynamic experiments, a zigzag path was chosen to test the system for dynamic movements, as shown in Figure 5. The zigzag path consisted of four-line segments, as shown. The zigzag path was chosen with sharp turns as it can replicate the field conditions better than a straight path. The line segment from station point 8 to station point 6 was divided into 12 intermediate points. Similarly, the second, third, and last line segment was divided by 9, 10, and 14 points, respectively. The coordinates of these points were found out using basic formulae of trigonometry. A worker carrying a tag was told to move on the path, as shown in the figure. At the same time, location data readings were recorded and compared with the already available survey data.

## 3. Results

### 3.1. Deployment Effort and Time Analysis

It was important to measure the man-minutes consumed for the various steps involved in the deployment and setup of the RTLS to quantify the level of effort required in the deployment of the system. Table 1 shows the man-minutes taken in completing steps in various attempts of RTLS deployment. The steps in the deployment of the system included the placement of bipods, which were used for surveying purposes to determine the position of the benchmarks using the laser scanner. Sensor stands placement included the placement of the stands that supported the sensors. The stands were folding-type stands, which were unfolded at the site, and the sensors were fixed to the stem of these stands using the screws.

**Figure 6:**
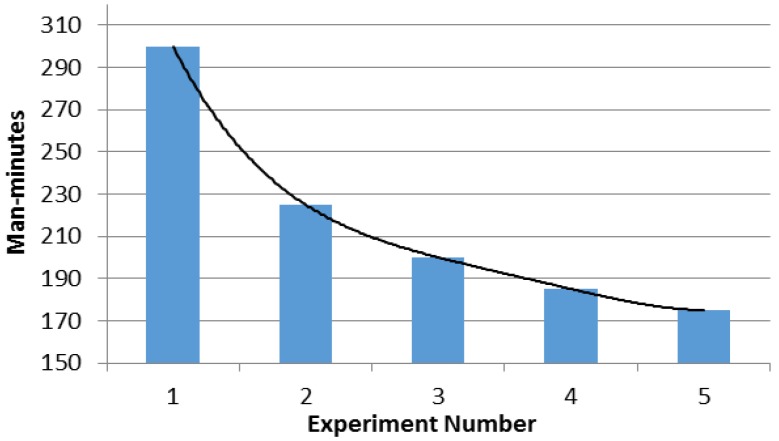
Learning curve over repeated deployments of the system.

Figure 6 shows the learning curve effect that can be observed. Total man-minutes consumed for the deployment started with 300 man-minutes and became stable after four repetitions, with a time duration of around 175 man-minutes.

### 3.2. Static Experiments

This section depicts a graphical representation of the accuracy results projected over the site area. It must be mentioned that different resolutions are used (i.e., the scale is not consistent across all figures) to highlight the relevant features and differences in each comparison set. Visual comparisons across the presented sets are not meaningful. The quantitative data used to plot graphical representations are available in Supplementary files.

#### Single Tag Versus All Tags Deployed at Once

**Figure 7:**
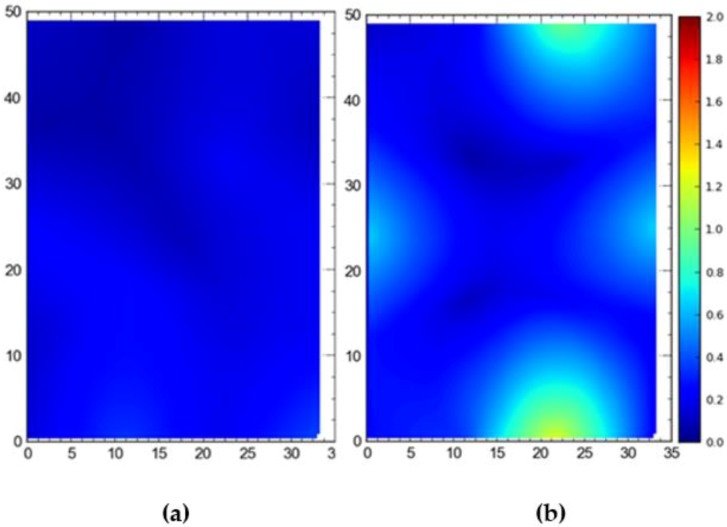
2D average accuracy comparison between (**a**) placing a single tag at different locations and (**b**) all tags tracked at once. Note: all measurements are in meters.

**Figure 8:**
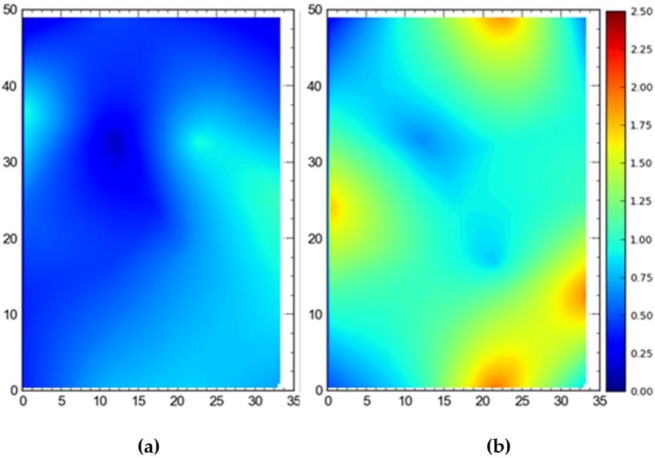
3D average accuracy comparison between (**a**) placing a single tag at different locations and (**b**) all tags tracked at once. Note: all measurements are in meters.

Figure 7 shows the 2D average accuracy (in meters) analysis for a single tag placed at different station points and 19 tags deployed at once on the test site. The mean values for both cases were 0.18 m and 0.36 m, respectively. Figure 8 depicts the 3D average accuracy analysis for a single tag placed at different station points and 19 tags deployed at once on the test site. Accuracy decreased as compared with the 2D average accuracy to 0.32 m and 0.6 m, respectively.

**Figure 9:**
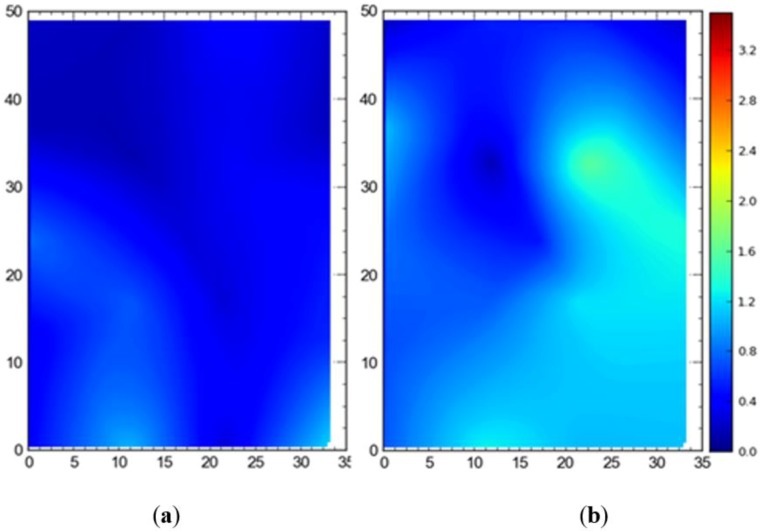
2D 95th percentile accuracy comparison between (**a**) placing a single tag at different locations and (**b**) all tags tracked at once. Note: all measurements are in meters.

**Figure 10:**
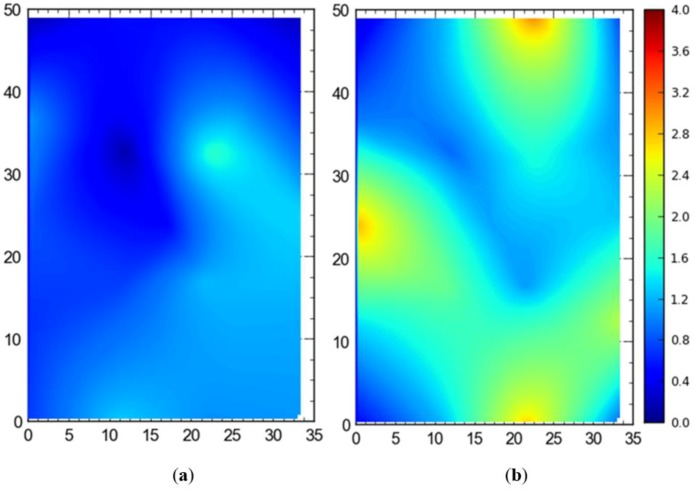
3D 95th percentile accuracy comparison between (**a**) placing a single tag at different locations and (**b**) all tags tracked at once. Note: all measurements are in meters.

Figure 9 shows the 95th percentile analysis (2D) for a single tag deployed all over the site and all of the station points occupied by the tags at once. Mean values were found to be 0.57 m for the single tag and 1.06 m for simultaneous deployment of all tags at once. Figure 10 depicts the 95th percentile analysis (3D) for the case when a single tag was deployed and moved around the site versus all tags deployed at station points simultaneously. The mean values were 0.82 m and 1.43 m, respectively.

1. Wired versus wireless setup

**Figure 11:**
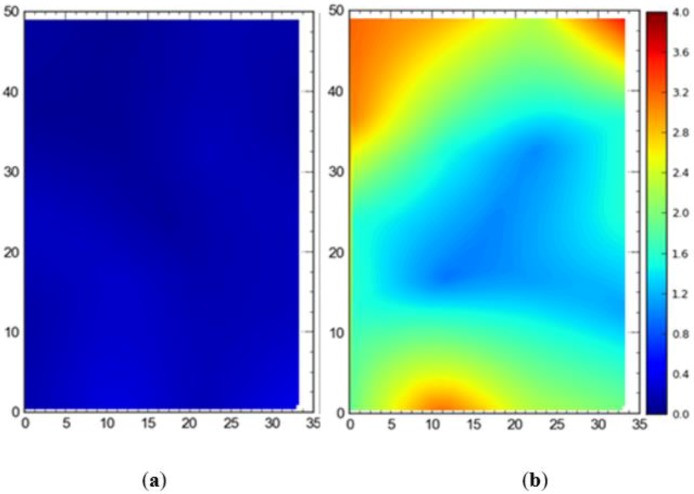
2D average accuracy comparison by placing tags at different locations at once in (**a**) wired and (**b**) wireless setups. Note: all measurements are in meters.

In order to investigate how accuracy in terms of DRMS and MRSE varies when both TDOA and AOA readings are used for localization and when only AOA readings are used, experiments were performed in the full site access configuration using a single tag deployed at different locations. Figure 11 shows the difference in accuracy (DRMS) between with TDOA and AOA only. The mean 2D accuracy was 0.18 m for the wired setup and 1.99 m for the wireless setup. Importantly, for the wireless setup, the accuracy is relatively good near the center of the site where the tag is being tracked by almost all of the sensors.

**Figure 12:**
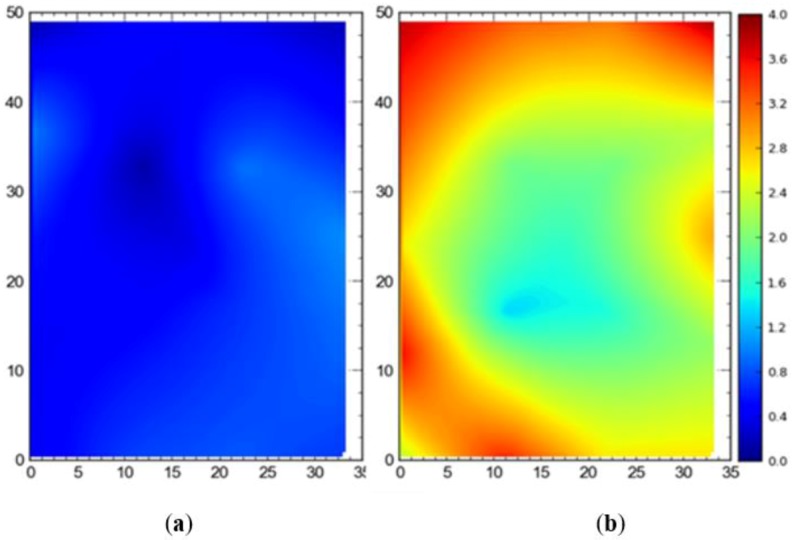
3D average accuracy comparison by placing tags at different locations at once in (**a**) wired and (**b**) wireless setups. Note: all measurements are in meters.

Figure 12 depicts the 3D accuracy difference in terms of MRSE for AOA only versus with TDOA readings. The results are similar to 2D results with mean values of 2.65 m and 0.57 m respectively. Again, the accuracy is better in the central region for AOA only readings.

2. Comparison between three deployment alternatives

**Figure 13:**
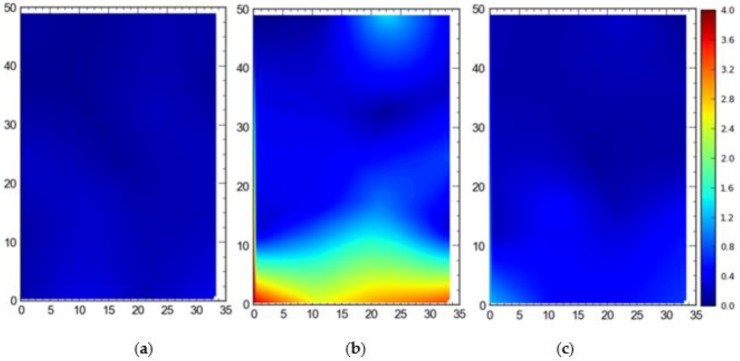
2D average accuracy comparison for the single tag deployed at all station points for the (**a**) full site access, (**b**) offsite setup, and (**c**) partial access. Note: all measurements are in meters.

Figure 13 shows the 2D average accuracy (DRMS) at the station points when a single tag was placed at all station points one by one. The mean accuracy was 18 cm, 102 cm, and 32 cm for the full site access, offsite setup, and partial access, respectively. The data were collected when the system was also using TDOA for the localization purpose.

**Figure 14:**
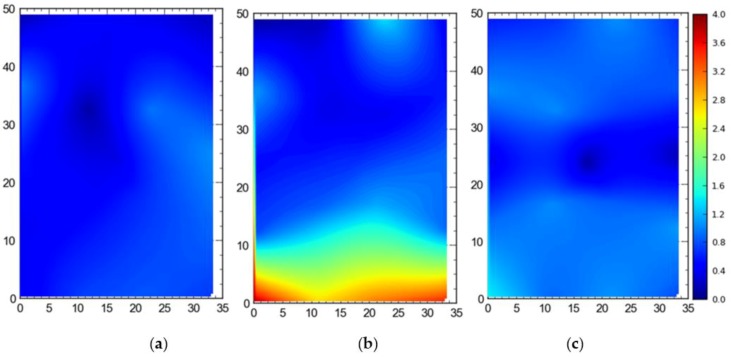
The 95th percentile accuracy comparison for the single tag deployed at all station points for the (**a**) full site access, (**b**) offsite setup, and (**c**) partial access. Note: all measurements are in meters.

Figure 14 shows the 95th percentile accuracy (DRMS) data for the different configurations when a tag was placed at all station points one by one. The mean values were recorded to be 57 cm, 124 cm, and 82 cm for the full site access, offsite setup, and partial access, respectively.

**Figure 15:**
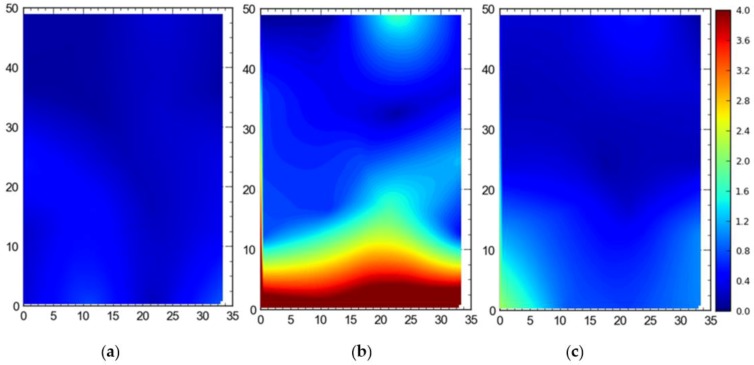
3D average accuracy comparison for the single tag deployed at all station points for the (**a**) full site access, (**b**) offsite setup, and (**c**) partial access. Note: all measurements are in meters.

Figure 15 shows the average accuracy for 3D accuracy (MRSE) for differcent configurations of the sensors of the RTLS. The mean values were recorded to be 32 cm for the full site access, 158 cm for the offsite setup, and 56 cm for the partial access.

**Figure 16:**
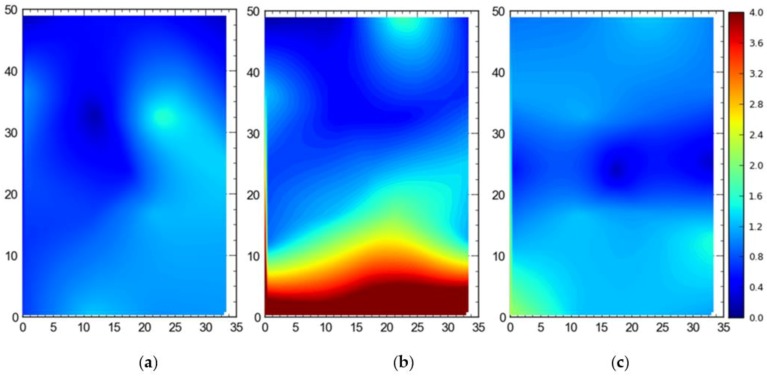
The 95th Percentile 3D accuracy comparison for the single tag deployed at all station points for the (**a**) full site access, (**b**) offsite setup, and (**c**) partial access. Note: all measurements are in meters.

Figure 16 shows the 95th percentile 3D accuracy (MRSE) data for the single tag deployed at all points. For the full site access, 82 cm was the mean value, while for the offsite setup and partial access, the mean values were measured to be 181 cm and 108 cm, respectively.

**Figure 17:**
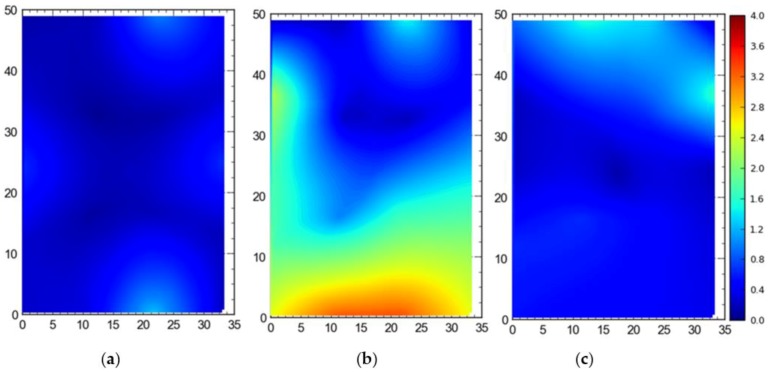
Average accuracy for the tags deployed at all station points simultaneously (2D) for the (**a**) full site access, (**b**) offsite setup, and (**c**) partial access. Note: all measurements are in meters.

Figure 17 shows the 2D average accuracy (DRMS) when all of the station points were occupied with the tags simultaneously. Accuracy dropped as compared with the single tag deployed at all points. The mean values were noted to be 38 cm, 144 cm, and 60 cm for the three configurations, respectively.

**Figure 18:**
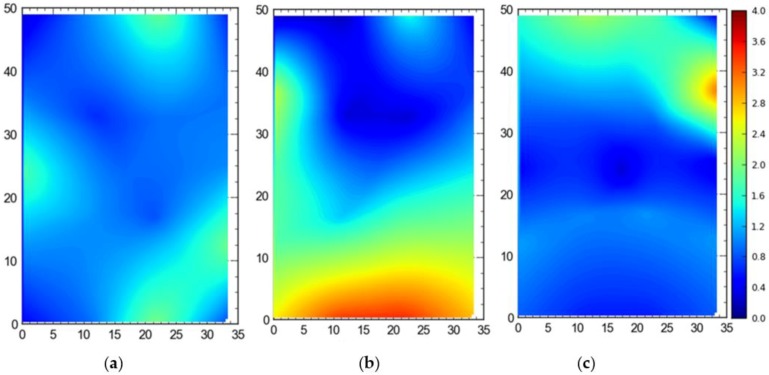
The 95th percentile accuracy for the tags deployed at all station points simultaneously (2D) for the (**a**) full site access, (**b**) offsite setup, and (**c**) partial access. Note: all measurements are in meters.

Figure 18 depicts the accuracy data for the 95th percentile 2D data when all of the station points were occupied with the tags. The mean values for the 95th percentile were 111 cm for the full site access, 155 cm for the offsite setup, and 106 cm for the partial access.

**Figure 19:**
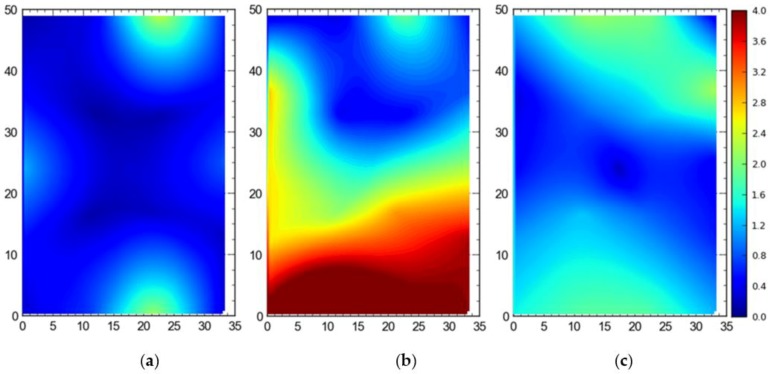
Average accuracy for the tags deployed at all station points simultaneously (3D) for the (**a**) full site access, (**b**) offsite setup, and (**c**) partial access. Note: all measurements are in meters.

Figure 19 shows the 3D accuracy data (MRSE) for the scenario when all of the station points were occupied by the tags simultaneously. The mean values were recorded to be 64 cm for the full site access, 231 cm for the offsite setup, and 114 cm for the partial site access.

**Figure 20:**
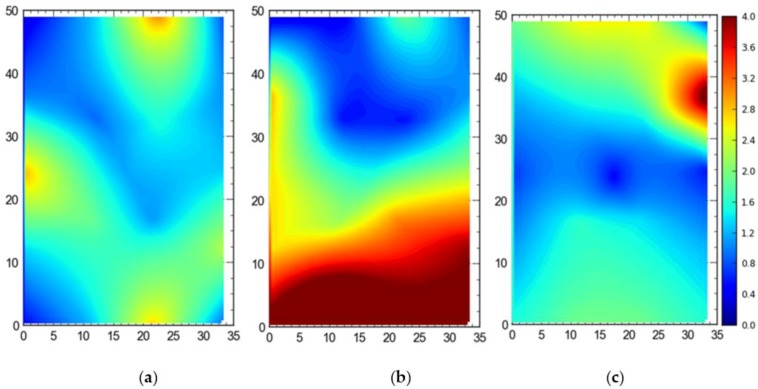
The 95th percentile accuracy for the tags deployed at all station points simultaneously (3D) for the (**a**) full site access, (**b**) offsite setup, and (**c**) partial access. Note: all measurements are in meters.

Figure 20 shows the 95th percentile 3D accuracy data (MRSE) for the case when all of the station points were occupied by the tags simultaneously. The mean values for the 95th percentile for the full site access, offsite setup, and partial access were 151 cm, 240 cm, and 158 cm, respectively.

### 3.3. Dynamic Experiments

**Figure 21:**
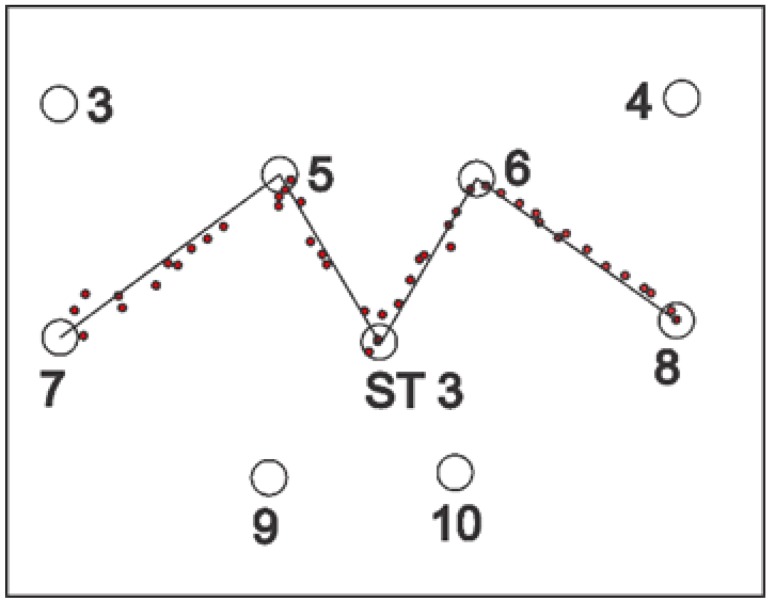
Accuracy for the dynamic experiment results for full site access.

Dynamic experiments were performed on the full site, off-site, and partial site access configurations. For full site access, the results showed an average accuracy of 77 cm based on DRMS. Visual representation of the experiment is shown in Figure 21. The station points are visible in the figure too. Location data are shown in the form of red circles as a person carrying the tag walked through the pre-determined path.

**Figure 22:**
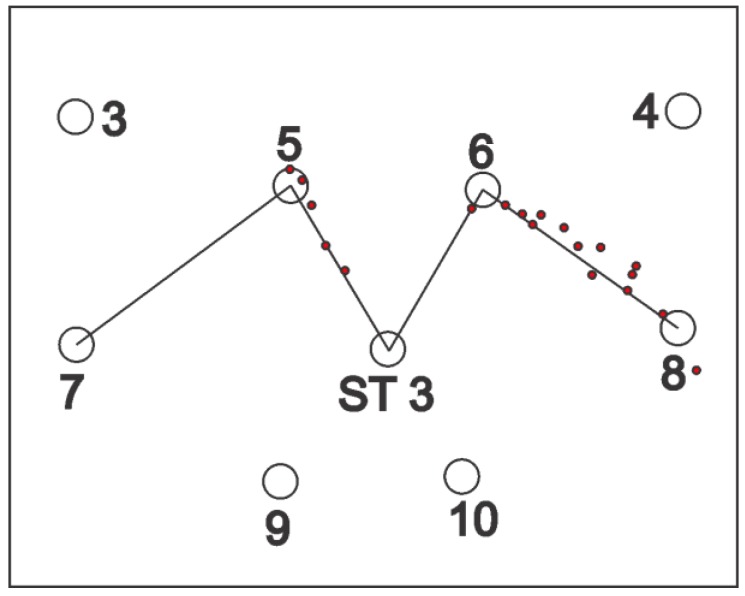
Dynamic experiment result for the offsite setup.

Similarly, the experiment was repeated for the offsite setup and average accuracy was calculated to be 146 cm. Figure 22 illustrates the experiment performed for the offsite setup.

**Figure 23:**
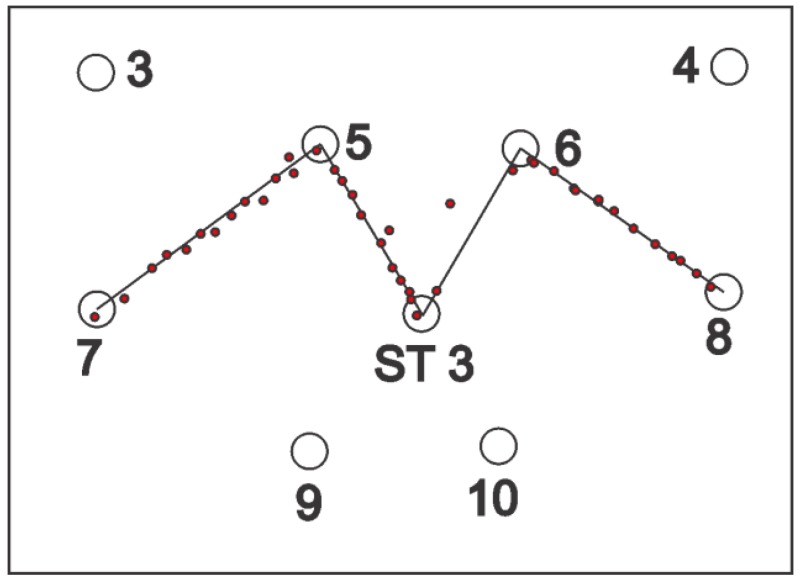
Dynamic experiment result for partial site access.

For partial site access, the average accuracy was calculated to be 117 cm for the 2D data. This accuracy was better than offsite setup, while it was less accurate than full site access. Figure 23 shows the experiment performed for partial site access.

## 4. Discussion

A series of experiments were conducted to study the behavior of the RTLS on a life-size scale site. The results of the tests show the variation in accuracy for the different experimental setups. For additional analysis and understanding of these experiments, heat maps were merged and overlaid with field of view (FOV) maps, as discussed in the following sections.

### 4.1. Static Experiments

The results indicate that average accuracy varied from a mean value of 0.18 m (for a single tag being tracked on site covered by full site sensors’ configuration in 2D, Figure 7a) to 2.3 m (for all tags deployed simultaneously using offsite sensors’ configuration in 3D, Figure 19b). Similarly, for the 95th percentile, the mean values were 0.35 m and 2.40 m for the respective setups. When compared with previous studies, this study achieved similar accuracy for single tag tracking in 2D and 3D for favorable environments, as Mok et al. [21] and Maalek and Sadeghpour [5] achieved. Similarly, the average accuracy for 2D tracking of a single tag is quite close to the accuracy achieved by Cho et al. [18] for open environment testing. A direct comparison of this study with Cheng et al. [17] was not possible because of the rather different experimental setups of the two studies. Besides, Saidi et al. [16] did not provide quantitative data and instead provided accuracy contour plots only, which hindered the comparison between the two studies.

It is evident that the deployment of all tags at once in the sensing area had a detrimental effect on the accuracy of the system (Figure 7, Figure 8, Figure 9 and Figure 10). This can be attributed to the number of tags being simultaneously tracked by the system. Besides, significant degradation in accuracy results from a wireless setup where only angle of arrival (AOA) information is being used for location estimation (Figure 11 and Figure 12). Closer observation reveals that the degradation can be overcome by increasing the number of sensors covering the area. The central region in Figure 11 and Figure 12, shaded in blue, represents the area of the jobsite that is adequately covered using AOA information only. However, getting the same accuracy over the entire site using AOA alone may prove prohibitively expensive owing to the costs associated with additional sensors. 

Moreover, the results show a significant difference between the average accuracy and 95th percentile results (Figure 7, Figure 8, Figure 9 and Figure 10). The results show a significant drop in the performance of the system when the stricter threshold of 95th percentile performance is applied. For example, Figure 7a shows a consistent accuracy of up to 0.5 m throughout the site area using a single tag, whereas the 95th percentile of the same data shows an accuracy of around 1 m, as seen in Figure 9a. This indicates that it is necessary to evaluate the system performance under stricter thresholds, such as the 95th percentile if the domain use is intended for the safety or security of assets and decision makers should not rely only on average accuracy.

**Figure 24:**
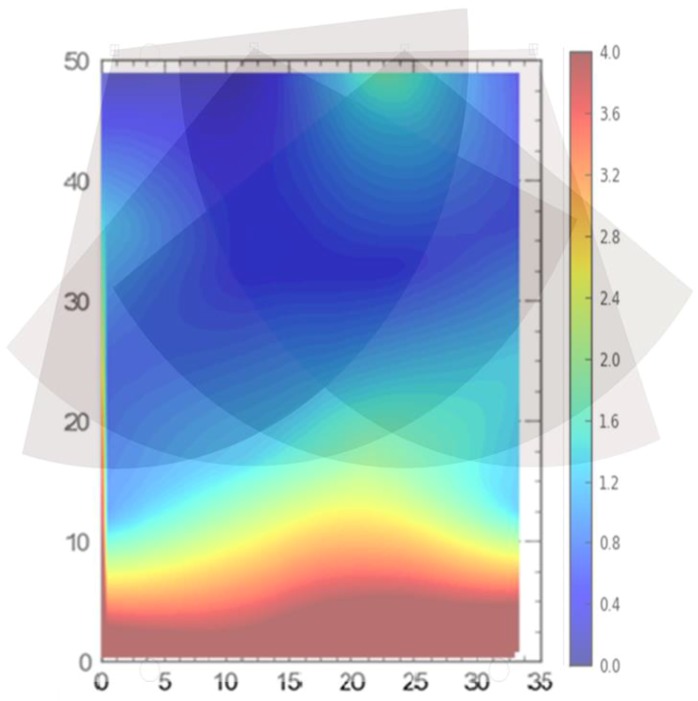
Heat map overlaid with sensors’ orientation (offsite setup).

For comparison between setups (i.e., full site, offsite, and partial access), it can be observed that the best average accuracy was obtained using full site access configuration, followed by partial access configuration (Figure 13, Figure 14, Figure 15, Figure 16, Figure 17, Figure 18, Figure 19 and Figure 20). It is important to note that the average accuracy for a setup is dependent on the configuration of the sensors. The worse average accuracy does imply that a configuration should not be used. Heat maps explain the phenomenon quite well. For offsite setup, the worst average accuracy was observed for all three scenarios, but this does not imply bad performance. For all of the experiments, the offsite setup showed worse performance around the boundary line joining the station points ST-4 and ST-5. If only the average accuracy is considered, it may be concluded that this configuration does not provide useful location data. If heat maps are seen, however, it could be noted that accuracy is much better near the center and the boundary joining the station points ST-1 and ST-2. Such a configuration is handy when access to the site is not available. Figure 24 shows the 95th percentile 3D heat map for a tag placed at all points one by one overlaid with the FOV of the sensors for the offsite setup. This overlaying explains much better accuracy in the middle than around the boundary line joining the station points ST-4 and ST-5. Nearer the sensors, the accuracy is much better, and the systems work best for the area that is covered by multiple sensors. As soon as the distance from the sensors is increased, the accuracy gradually starts to drop.

**Figure 25:**
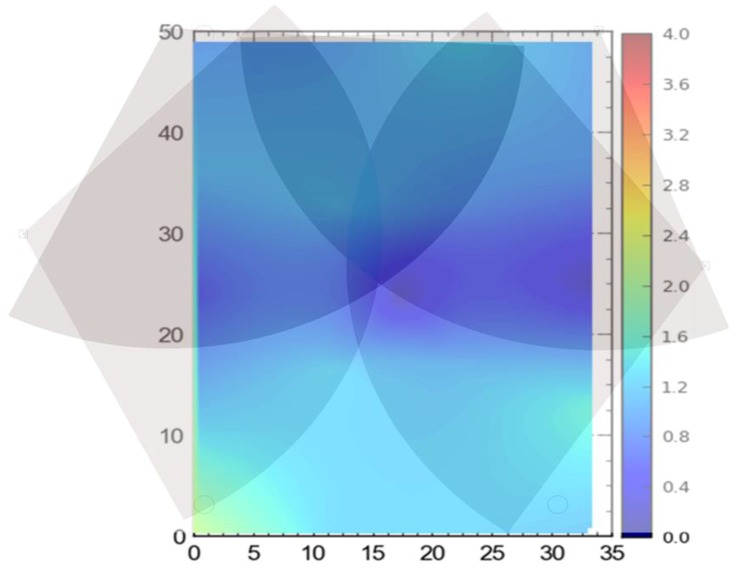
Heat map overlaid with sensors’ orientation (partial access).

Similarly, for partial site access, much better accuracy is observed in the central region than other parts of the site. Figure 25 shows the 95th percentile heat map for the single tag deployed at all points for 3D overlaid with the sensors FOV. This overlaid map explains well why the accuracy is much better in the central region than the rest of the site. Similarly, it also explains why the areas around the boundary joining station points ST-4 and ST-5 have lower accuracy as compared with the rest of the site.

### 4.2. Dynamic Experiments

**Figure 26:**
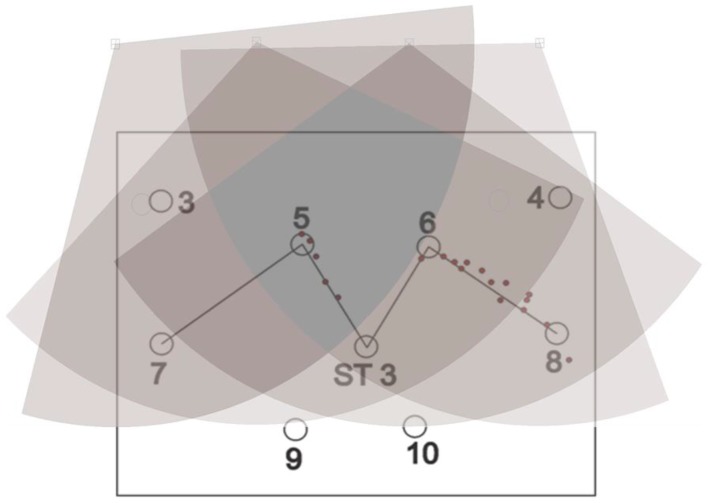
Dynamic experiment results overlaid with sensors’ orientation (offsite setup).

Sub-meter accuracy for the full site access configuration and around 1 m accuracy for the partial site access configuration demonstrate good results for real-time tracking of the construction resources. In contrast, the offsite setup showed decreased average accuracy of around 1.5 m. Importantly, for the offsite setup, a problem can be seen that the system did not display the location data for the line segment from station points 5 to 7 and 6 to ST-3 (Figure 22). Graphical representation (Figure 26) of the results of dynamic experiment overlaid with sensors’ orientation explains this problem well.

When the tag carrier moved from station point 8 to 6, the tag was visible to the sensors. However, when he moved from station point 6 to 3, the tag was not visible to the sensors as the signals were blocked by the worker carrying the tag as the tag was being carried in the hand. The case was similar when the carrier moved from station point 5 to 7. This phenomenon of signal interference is also pointed out by many researchers [19,20,27,44]. Therefore, the authors suggest that, while using the RTLS, the tags should be elevated by attaching them to the hard hats of the workers, so that they can be sighted by the sensors without any obstruction.

### 4.3. Practical Recommendations

On the basis of the accuracy parameters obtained, man-minutes requirement, and hands-on experience on the RTLS, the authors conclude that deployment of UWB-based RTLS may not be feasible for all construction jobsites. Safety implications resulting from cables and the safety of the RTLS system itself can outweigh the benefits that the system may bring to the jobsite. However, the system is highly recommended for critical project activities, for example, critical crane lifts, girder launching of a flyover, concrete pouring of a critical slab, erection of important steel structures, fabrication yards, and offsite fabrication. For such deployment, the following recommendations are made to enable a successful setup with increased accuracy: (1) deploy the sensors outside the boundary of the area to be monitored by 5 m approximately, in order to ensure robust monitoring even around the boundary of the area; (2) fix the sensors on firm supports such as walls to improve accuracy, as vibrations and wind may affect the sensors installed on the tripods; (3) keep the sensors as high as possible (preferably more than 2 m); (4) if enhanced accuracy is required, use the wired sensors setup and provide site coverage using multiple sensors; (5) if the wired setup is not possible, accuracy for the wireless setup can be enhanced by increasing the number of the sensors; (6) keep on changing the setup (in term of location and position of the sensors), as the characteristics of the construction site change during the lifecycle of the project; (7) tags should be attached to the top of the resources such that sensors may have direct line of sight; and (8) tag the wires according to the deployment needs, as this will help in repeated deployment of the system.

## 5. Conclusions

UWB-based RTLS has been successfully used in the manufacturing industry, where the system is deployed permanently, calibrated once, and then re-calibrated only when needed, and where wiring can be managed with the fixed infrastructure of the setup. However, construction sites are dynamic and do not allow for a permanent deployment of these systems. The experiments recorded the man-minutes required for each set up as an indicator of the level of effort necessary to get beneficial use of this system on construction jobsites. The actual effort on a particular site will be influenced further by the existing jobsite conditions and access requirements.

Multiple experiments were setup to explore and investigate the feasibility of UWB-based RTLS for construction. The results reveal that using 2D tracking instead of 3D, tracking a lesser number of tags, using a wired setup instead of a wireless setup, and providing site coverage from multiple directions enhances the accuracy of tracking. Furthermore, ideal deployment conditions provide fair enough coverage of the construction site, but seem to be difficult to achieve on actual jobsites. Additionally, settings such as the offsite setup may help by location tracking of the construction assets without the deployment of the RTLS infrastructure on construction jobsite, but will provide only a limited coverage area. Full site access and adding additional sensors will enhance the coverage and accuracy of the RTLS, but at the cost of more cabling on traditionally dynamic construction environment, which might increase safety hazards. Importantly, the study has highlighted that the accuracy of UWB-based RTLS varies greatly based on the definition (i.e., from 2D average accuracy to 3D 95th percentile) and based on the RTLS infrastructure (i.e., what type of access is available to the site and whether or not wired cables can be deployed on site). Consequently, such information is useful to know the requirements for the desired accuracy for the RTLS.

While this study has added to the body of knowledge related to the use of UWB-based RTLS for construction sites, there are a few limitations of this study too, which further studies should investigate. For example, this study should be repeated on an actual construction site throughout the duration of the project. Secondly, future studies should also explore how UWB-based RTLS could be used along with other safety prevention methods to make construction sites even safer. For instance, risk-taking behavior of construction is common and is a cause of construction accidents [45,46]. Studies may explore how UWB-based RTLS could be used to compliment behavior-based safety to reduce such risk-taking tendencies. Similarly, UWB-based RTLS could be used in combination with real-time physiological data of construction workers [47], in order to enhance occupational safety on construction sites. Last, but not the least, by combining location data with ergonomic analysis [48,49], work conditions could be improved further by identifying the more critical work stations on the site.

## Figures and Tables

**Table 1:** Man-minutes recorded for each deployment of the experimental setup in the experiment location.

Time Component	Experiment Number				
	1	2	3	4	5
1. Bipods placement	15	10	10	10	10
2. Sensors stands placement	15	15	10	15	15
3. Layout and cabling	100	100	40	60	50
4. Orientation and survey	80	60	80	80	60
5. System calibration	90	40	60	20	40
Cumulative time (man-minutes)	300	225	200	185	175

## Supplementary Materials

The following are available online at https://www.mdpi.com/1660-4601/17/7/2219/s1, Quantitative data for the figures Figure 7, Figure 8, Figure 9, Figure 10, Figure 11, Figure 12, Figure 13, Figure 14, Figure 15, Figure 16, Figure 17, Figure 18, Figure 19 and Figure 20.
